# Analysis of Assessment Methods for Detecting Nicotine Residue and Its Impact on Humans: A Systematic Review

**DOI:** 10.3390/ijerph22040621

**Published:** 2025-04-16

**Authors:** Audrey A. Almassi, Brian G. G. Oliver, Sheree M. Smith

**Affiliations:** 1 Faculty of Science, School of Life Sciences, University of Technology Sydney (UTS), Ultimo, NSW 2007, Australia; brian.oliver@uts.edu.au; 2 Respiratory Cell and Molecular Biology Group, Woolcock Institute of Medical Research, Macquarie Park, NSW 2113, Australia; sheree.smith@adelaide.edu.au; 3 Faculty of Health and Medical Sciences, Adelaide Nursing School, University of Adelaide, Adelaide, SA 5000, Australia

**Keywords:** nicotine, thirdhand smoke, residue, contamination, assessment method, human

## Abstract

Introduction: Thirdhand smoke (THS) was first identified by Graham and colleagues in 1953, and nicotine was detected in household dust from smokers in 1991. Thirdhand smoke (THS) consists of toxic nicotine residues that persist on surfaces long after tobacco use, posing a significant public health concern. Individuals can be exposed to thirdhand smoke through skin contact or inhalation, particularly affecting children and infants who are most vulnerable to tobacco contaminants. This review aims to assess the effectiveness of different methods for measuring nicotine THS residues to evaluate their accuracy across various age groups. Methods: Relevant literature was sourced from databases including ProQuest (Ovid), Medline (Ovid), Embase (Ovid), Scopus, and the Cochrane Library. The timeframe for included studies ranged the last 25 years, from 1999 to 2024. Eligible participants consisted of human populations exposed to thirdhand smoke residue. For this review, the animal studies were excluded. There were no restrictions regarding age, sex, ethnicity, or nationality for participant selection. For data management and screening, the Covidence systematic tool was utilized. Data extraction was performed independently by two reviewers. This protocol was registered with PROSPERO (CRD42024574140). Results: A total of 394 studies were retrieved from 5 databases for the initial screening. A total of 67 studies included in full-text screening, and ultimately, 36 studies were selected for full review. The studies were classified into four categories based on assessment methods: (1) analysis of human secretions, including salivary or urinary tests; (2) cellular analysis utilizing cellulose substrates or paper-based materials; (3) environmental assessments, which examined outdoor surfaces, vehicles, residential spaces, and fabrics; and (4) epidemiological assessments, employing surveys or questionnaires. Non-invasive matrices such as saliva and urine were frequently utilized for biomarker analysis. The studies collectively investigated nicotine and its metabolites in human biological samples, environmental surface contamination, and thirdhand smoke (THS) exposure. They employed a diverse range of assessment tools including surveys, machine learning technique, and cellulose-based substrates. Conclusions: This review identified various selective testing methods for detecting thirdhand smoke (THS) from nicotine. These assessment methods have advantages and disadvantages and underscores the need for further research. Improving these techniques for assessment of THS could significantly improve our understanding of the impact THS has on human health.

## 1. Introduction

Despite the well-documented risks associated with tobacco use, it remains one of the leading causes of mortality worldwide. In 2019, over one billion people were regular tobacco users. Annually, tobacco-related illnesses result in approximately 8.7 million deaths among active smokers (first-hand exposure) and over 1 million deaths among passive smokers (second-hand exposure) [1,2]. Globally, smoking accounted for 20.3% of deaths among males and 5.8% among females [3]. Smoking is a major cause of various diseases, including cardiovascular diseases, respiratory disorders, and cancer. Tobacco smoke contains a blend of 7000 chemical substances emitted from tobacco products. First-hand smoke, also known as mainstream smoke, refers to the smoke directly inhaled by a person using tobacco products such as cigarettes, vapes, or pipes [4]. In contrast, second-hand smoke (also called passive or environmental tobacco smoke) refers to smoke that is exhaled by smokers or released from tobacco products and involuntarily inhaled by others [5]. Studies have shown that second-hand smoke, a mixture of mainstream and side-stream smoke, is more toxic than mainstream smoke alone due to its higher concentrations of harmful chemical compounds [6]. Reports on the impacts of second-hand smoke (SHS) have been available since 1928 [7]. This type of smoke consists of approximately 85% side-stream smoke and 15% mainstream smoke. Second-hand smoke can persist in the environment for 2–3 h after tobacco use [8].

Tobacco use, including cigarette smoking, vaping, and waterpipe smoking, is associated with numerous health problems, such as heart attacks, cancer, respiratory diseases like pneumonia, and weakened immune function [9]. People may be exposed to these pollutants in various environments, including homes, vehicles, and public spaces [10].

In 1953, Graham and his colleagues discovered thirdhand smoke (THS) [11]. A subsequent study in 1991 reported the presence of nicotine residues in the dust of smokers’ homes [12]. Thirdhand smoke refers to persistent residues or aged second-hand smoke that accumulates, penetrates, or adheres to surfaces, curtains, furniture, or clothing. These residues can remain for extended periods after tobacco use, even after cleaning [13].

Nicotine, a potent alkaloid predominantly found in tobacco, is a major factor in smoking addiction due to its structural resemblance to acetylcholine, a crucial neurotransmitter in the brain [14]. In the context of thirdhand smoke (THS), nicotine is particularly significant because of its associated health risks, chemical reactivity, and prolonged environmental persistence [15]. As the primary addictive agent in tobacco smoke, nicotine not only drives dependence but also acts as a precursor to toxic compounds formed through its interactions with environmental elements [15]. After smoking, nicotine residues persist on indoor surfaces, where they undergo chemical transformations through reactions with indoor oxidants such as ozone and nitrous acid [16]. For example, nicotine’s interaction with ozone leads to the formation of secondary organic aerosols (SOA) and other toxic byproducts, including cotinine, which is associated with mutagenic and carcinogenic effects [16]. The heterogeneous oxidation of nicotine generates these hazardous compounds, posing significant health risks, particularly in indoor environments where prolonged exposure is more likely [16].

**Figure 1:**
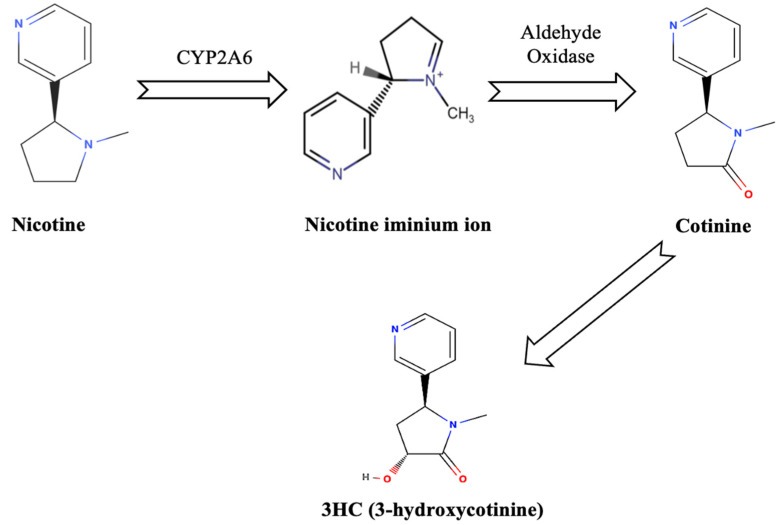
Nicotine metabolism.

Cotinine and 3HC (3-hydroxycotinine) are formed when nicotine is oxidized by the enzyme cytochrome P450 2A6 in the human liver [17]. This enzyme converts nicotine into a nicotine iminium ion, which is then acted upon by aldehyde oxidase, ultimately producing these metabolites as shown in Figure 1 [17].

Tobacco product residues such as nicotine can linger in vehicles or homes where active smoking has occurred [18]. Indoor environments can harbor multiple pollutants from tobacco smoke, classified as first-hand, second-hand, and thirdhand smoke. Specific data on indoor concentrations of first-hand smoke are limited since it is directly inhaled by smokers [4]. However, it is well established that active smoking introduces high levels of harmful chemicals into the immediate environment. Indoor concentrations of second-hand smoke pollutants vary based on factors such as ventilation and smoking frequency. For example, nicotine levels in indoor air due to SHS have been reported 26.92 μg/m^3^ [19]. Particulate matter (PM2.5) concentrations in primary smoking areas can reach 84 μg/m^3^, with adjacent areas experiencing levels around 63 μg/m^3^ [4]. Studies on thirdhand smoke have detected nicotine concentrations on indoor surfaces ranging from 0.7 to 1.9 μg/m^2^ in areas where smoking has occurred [4].

Individuals are not entirely safe from THS and can be exposed to thirdhand smoke residue through dermal absorption, non-dietary ingestion pathways, or inhalation [13]. Children and infants are particularly vulnerable to tobacco chemical contaminants because they often touch contaminated surfaces [13]. It has been reported that chemical compounds in thirdhand smoke can be released into the air as they age or react with nitrous acid or ambient oxidants, forming secondary pollutants [20].

Current techniques for detecting thirdhand smoke predominantly rely on analysing nicotine, cotinine and 3HC (3-hydroxycotinine), which are the main metabolites of nicotine, as markers of contamination [21].

In research on thirdhand smoke (THS), nicotine is frequently emphasized due to its high concentration and reactive nature. Nicotine residues can persist on surfaces within indoor environments long after active smoking has ended, thereby extending the duration of exposure [22]. Furthermore, nicotine is chemically reactive with common indoor pollutants, such as nitrous acid, resulting in the formation of carcinogenic tobacco-specific nitrosamines (TSNAs), including NNK and NNN [22]. These chemical transformations underscore the critical role of nicotine in contributing to the overall toxicity of THS.

It is acknowledged, other compounds are present in THS, such as polycyclic aromatic hydrocarbons (PAHs) and heavy metals, exhibit different chemical behaviours however, devices that simulate smoking, such as vapes, the vape liquid predominantly contain nicotine. Other compounds such as PAHs, are less volatile and adhere strongly to surfaces, reducing their likelihood of re-emission into the air or participation in chemical reactions [22]. Furthermore, primary aromatic amines (PAAs) are semi-volatile and highly reactive organic compounds capable of high interaction with proteins and DNA, leading to mutagenic effects and contributing to carcinogenesis [23]. These compounds are frequently detected in tobacco smoke and its related products. In indoor environments, PAAs are introduced primarily through the deposition of dust originating from waterpipe and cigarette smoke [23]. Heavy metals are non-volatile and remain bound to particulate matter, which limits their mobility and potential for interaction with other indoor pollutants [22]. Studies have identified over 20 compounds that were greater in dust from houses of smokers including nomicotyrine and 3-ethenylpydridine [24] which had not previously been reported in house dust as they utilized previous study samples [25].

As a result, these substances may pose a lower risk of secondary exposure compared to nicotine. The prioritization of nicotine in THS research is justified by its abundance, chemical reactivity, and ability to generate secondary toxic compounds, all of which significantly influence the toxicity of indoor environments and the associated health risks. Additionally, Richardot and his colleagues [24] report that nicotine has been identified as having the highest potential for developmental toxicity.

Identifying chemical biomarkers of tobacco is crucial for evaluating the health impacts of exposure to second-hand smoke (SHS) and thirdhand smoke (THS) [26]. The measurement of cotinine, the primary biomarker of nicotine, in urine, blood, and saliva is a widely used method to assess the type, level, and frequency of exposure to tobacco products and smoke, owing to its extended half-life [26].

Furthermore, hair can serve as a non-invasive matrix for assessing thirdhand smoke (THS) exposure, potentially offering advantages over other methods by being less influenced by human body metabolism [26]. Another method for assessing THS exposure is collecting hand wipes, usually used in toddlers to measure THS exposure [27]. While there is no definitive marker for thirdhand smoke (THS) exposure, nicotine levels on the hands can act as an indirect measure, as THS pollutants in non-smoking environments can be absorbed through the skin [27].

A comprehensive systematic review by Díez-Izquierdo et al. focused on thirdhand smoke [28]. The review evaluated various biomarkers of THS exposure, examined research involving cell, animal, and human models, and explored related policies and social beliefs. However, the study did not categorize the assessment methods and included research papers that analysed various chemical components beyond nicotine and its metabolites.

Our systematic review aims to identify the measurement methods utilized to detect thirdhand nicotine smoke, examine their target populations, and evaluate the accuracy of these methods in detecting thirdhand smoke residues, including nicotine, cotinine, and 3-hydroxycotinine (3HC). Furthermore, this review highlights areas for further research in existing research and suggest directions for future studies on the health effects of thirdhand nicotine smoke exposure.

## 2. Methods

### 2.1. Registration and Guidelines

This systematic review was registered in the International Prospective Register of Systematic Reviews (PROSPERO) registry (CRD42024574140). This review follows the Preferred Reporting Items for Systematic Reviews and Meta-Analysis (PRISMA) 2020 guidance [29].

### 2.2. Search Strategy

A search was conducted on 5 databases (Medline (Ovid), ProQuest (Ovid), Embase (Ovid), Scopus and Cochrane Library), including studies published within the last 25 years (1999–2024).

The key search terms were identified using “Thirdhand”, “tobacco”, “nicotin*”, “smok*”, “product”, “contamin*”, “residu*” and “test*” (Tables S1–S5). These terms were truncated, and Boolean operators were used to ensure the capturing of all necessary data. Also, the first author (A.A.) performed manual reference list screening for additional studies from identified published studies.

### 2.3. Paper Selection

The inclusion criteria for this systematic review are as follows: [1] all studies included samples or participants which were exposed to tobacco product metabolites such as nicotine, cotinine and/or 3HC (3-hydroxycotinine), [2] participants can be in any age range (adults, elders, children including infants, toddlers, and adolescents), and [3] detecting the tobacco or tobacco residue on hands was this study’s outcome. Studies with animal models, review articles, pilot studies, the grey literature with incomplete results, study protocols and non-English publications were excluded.

In accordance with the Cochrane Reviews Handbook [30] the consistency between authors during the screening phase is crucial for the reliability and validity of the review findings. To facilitate this process, Covidence systematic tool was used for the management and screening of all data.

After removing the duplicates using Covidence systematic tool, two researchers (A.A. and S.S.) independently screened the titles and abstracts of the full-text studies.

Any conflicts and disagreements were resolved by the third researcher (BO). For the next phase of this review, two researchers (A.A. and S.S.) reviewed the full-text articles to assess their eligibility. Any conflicts and disagreements were resolved by the third researcher (BO).

### 2.4. Data Extraction

Two researchers (A.A. and S.S.) extracted data from the eligible articles using a review-specific data extraction sheet (Table S6). The following data were extracted: authors, publication year, country, target population, biological matrix or sample, biomarkers, number of participants and category of assessment method. The data in this systematic review investigated the type of tobacco metabolites found in human biological matrices, skin, and environments, such as communal areas and cell cultures. Also, questionnaires were utilized in some studies to collect data on THS exposure, sociodemographic status of individuals and household characteristics such as living in multiunit housing or living with smoker family members.

## 3. Results

**Figure 2:**
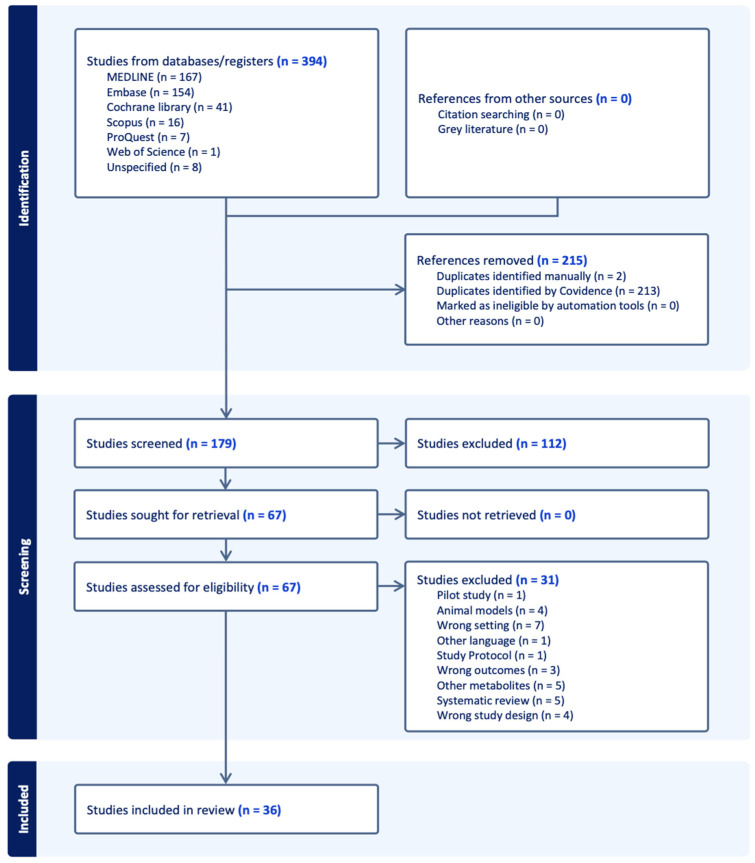
PRISMA flow diagram.

The PRISMA flow diagram is presented in Figure 2, representing the literature search retrieval process and the number of studies identified at each stage of the review process. From the total number of 394 studies from selected databases, 215 studies were removed and excluded. After screening the title and abstract for eligibility, 179 studies remained, of which 112 irrelevant studies were identified and excluded. A total of 67 studies were retrieved for full-text review, and 31 studies were excluded due to different reasons such as wrong setting, pilot study, wrong outcomes or animal models in this study. Thirty-six studies were eligible to be included in this systematic review and data extraction.

### 3.1. Study Characteristics

The included studies were published between 1999 and 2024 and reported in Table 1. A total of 11,116 human participants were included across the 16 studies, with sample sizes ranging between 5 and 5296 participants [13,31,32,33,34,35,36,37,38,39,40,41,42,43,44,45,46]. A total of 7 studies provided data on the number of fabrics analysed, with sample sizes in some studies ranging from 4 to 12 [20,47,48,49,50,51,52]. Two studies utilized cellulose papers for determination of presence of nicotine residues in cells [53,54]. Seventeen studies conducted questionnaires, surveys, interviews and machine learning to evaluate of thirdhand smoke as well as utilizing other measurement methods [18,25,31,33,34,36,37,39,42,43,44,45,55,56,57,58].

Twenty-five studies were conducted in the USA [13,18,25,31,32,33,35,36,37,38,39,40,41,42,44,45,47,48,49,51,52,54,56,57,59], three in European countries such as Spain, Hungary and Belgium [43,49,60], three in Korea [34,46,58], one in Canada [55], one in Iran [50], two in the UK [61,62], one in Brazil [53] and one in Taiwan [20].

Participants were mixed of adults who were exposed to nicotine thirdhand smoke or being a smoker, infants, toddlers or adolescents who were exposed to thirdhand smoke and live with a smoking family member or neighbour.

Majority of studies used mass spectrometry techniques such as different types of liquid chromatography, gas chromatography or wipes to measure and determine the type of biomarkers in the samples.

**Figure 3:**
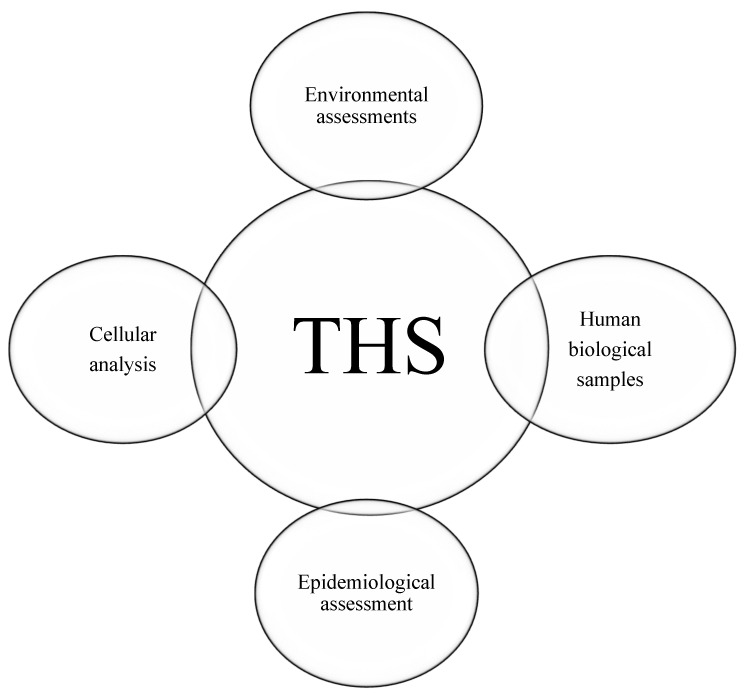
Four categories of THS detection samples.

After analysing the studies, four categories of assessment methods were identified: (1) human biological samples, (2) cellular analysis, (3) environmental assessments, and (4) epidemiological analysis (Figure 3).

**Table 1:** Study characteristics table.

Title of the Study	Author(s)	Year of Publication	Country	Target Population	Biological Matrix or Detection Samples	Biomarkers	Number of Participants	Category of Assessment Method(s)
A simple and rapid method for the determination of nicotine in third-hand smoke by liquid chromatography and its application for the assessment of contaminated outdoor communal areas	Samira Inácia Santos e Silva, Paul Bowdler, Danielle Giltrow, Stephanie Riddell and Kevin C. Honeychurch [61]	2015	UK	Individuals in outdoor communal areas, particularly those near public entrance ways, where thirdhand smoke (THS) contamination can occur due to smoking activities	Dust-wipe samples	Nicotine	N/A	Hydrophilic interaction liquid chromatography (HILIC) and UV detection/ Environmental assessments
Adhesion and Removal of Thirdhand Smoke from Indoor Fabrics: A Method for Rapid Assessment and Identification of Chemical Repositories	Giovanna L. Pozuelos, Peyton Jacob, Suzaynn F. Schick, Esther E. Omaiye and Prue Talbot [47]	2021	USA	Both smokers and non-smokers who are exposed to THS	Indoor fabrics/upholstery cotton, terry cloth, upholstery polyester, and wool carpet	Nicotine; myosmine; 2,39-bipyridine; cotinine; N-formylnornicotine; nicotelline; NNN; NNK; NNA	4	LC–MS/MS, fluorescence intensity, HPLC–(high-performance liquid chromatography), autofluorescence/ Environmental assessments
Assessing second-hand and thirdhand tobacco smoke exposure in Canadian infants using questionnaires, biomarkers, and machine learning	Jaclyn Parks, Kathleen E. McLean, Lawrence McCandless, Russell J. de Souza, Jeffrey R. Brook, James Scott, Stuart E. Turvey, Piush J. Mandhane, Allan B. Becker, Meghan B. Azad, Theo J. Moraes, Diana L. Lefebvre, Malcolm R. SearsPadmaja Subbarao and Tim K. Takaro [55]	2021	Canada	Infants	Urine, machine learning	Cotinine, trans-3′-hydroxycotinine (3HC)	N/A	Liquid chromatography–atmospheric pressure chemical ionization tandem mass spectrometry. Machine learning/ Epidemiological assessment
Cellular effects of thirdhand tobacco smoke from smokers homes	Luciana Rizzieri Figueiró, Rafael Linden, Ana Luiza Ziulkoski and Denise Conceição Mesquita Dantas [53]	2017	Brazil	Adults	Cellulose paper, questionnaire	Nicotine	12	Questionnaire, gas chromatography–mass spectrometry (GC–MS), cell treatment and cytotoxicity assays/ Cellular analysis
Changes and stability of hand nicotine levels in children of smokers: Associations with urinary biomarkers, reported child tobacco smoke exposure, and home smoking bans	Georg E. Matt, Ashley L. Merianos, Lara Stone, Chase Wullenweber, Penelope J.E. Quintana, Eunha Hoh, Nathan G. Dodder, Nicolas Lopez Galvez, E. Melinda Mahabee-Gittens [31]	2023	USA	Children (0–11 yrs old)	Hand wipes, urine, survey	Nicotine, cotinine, trans-3′-hydroxycotinine, nicotelline N-oxides, and 4-(methylnitrosamino)-1-(3-pyridyl)-1-butanol	129	Electronic survey assessments/liquid chromatography–tandem mass spectrometry (LC–MS/MS)/ Human biological samples assessment
Comparative study of comprehensive gas chromatography–nitrogen chemiluminescence detection and gas chromatography–ion trap–tandem mass spectrometry for determining nicotine and carcinogen organic nitrogen compounds in thirdhand tobacco smoke	Noelia Ramírez, Laura Vallecillos, Alastair C. Lewis, Francesc Borrull, Rosa M. Marcé, Jacqueline F. Hamilton [62]	2015	UK	Non-smokers who are exposed to THS including infants	Dust sample	Nicotine (16 organic nitrogen carcinogens found in tobacco smoke, such as aromatic amines, nitrocompounds, N-nitrosamines, tobacco-specific nitrosamines, and nicotine as a tobacco marker)	18	Gas chromatography coupled to ion trap mass spectrometry detection; two-dimensional gas chromatography coupled to a nitrogen chemiluminescence detector (GC–× GC–NCD)/ Environmental assessments
Contamination of surfaces in children’s homes with nicotine and the potent carcinogenic tobacco-specific nitrosamine NNK	Ashley L. Merianos, Georg E. Matt, Timothy M. Stone, Roman A. Jandarov, Eunha Hoh, Nathan G. Dodder, Penelope J. E. Quintana, Nicolas Lopez-Galvez, Lara Stone and E. Melinda Mahabee-Gittens [32]	2023	USA	Children who live with smokers	Surface wipe samples, urine samples	Nicotine, cotinine	84	Questionnaire, LC– MS–MS/ Human biological samples assessment, epidemiological assessment
Cotton pillows: A novel field method for assessment of thirdhand smoke pollution	Georg E. Matta, Eunha Hohb, Penelope J.E. Quintanab, Joy M. Zakarianc, Jayson Arceo [48]	2018	USA	Former smokers and their homes	Cotton pillow	Nicotine	12	Isotope-dilution liquid chromatography–tandem mass spectrometry/ Environmental assessments
Detection of nicotine as an indicator of tobacco smoke by direct analysis in real time (DART) tandem mass spectrometry	Akos Kuki, Lajos Nagy, Tibor Nagy, Miklos Zsuga, Sandor Keki [49]	2014	Hungary	Smokers, non-smokers, and individuals exposed to second-hand and thirdhand smoke	Clothes, cups	Nicotine	N/A	Direct analysis in real time (DART) mass spectrometry/ Environmental assessments
Detection of thirdhand smoke on clothing fibres with a surface acoustic wave gas sensor	Chi-Yung Cheng; Shih-Shen Huang; Chia-Min Yang; Kea-Tiong Tang; Da-Jeng Yao [20]	2016	Taiwan	Individuals exposed to thirdhand smoke	Clothing fibres	Nicotine	N/A	Surface acoustic wave (SAW) gas sensor/ Environmental assessments
Differential associations of hand nicotine and urinary cotinine with children’s exposure to tobacco smoke and clinical outcomes	E. Melinda Mahabee-Gittens, Ashley L. Merianos, Roman A. Jandarov, Penelope J.E. Quintana, Eunha Hoh, Georg E. Matt [33]	2021	USA	Children/Parents	Hand wipes, urine, questionnaire	Nicotine	276	LC–MS/MS, questionnaires/ Human biological samples assessment, epidemiological assessment
Electronic cigarette chemicals transfer from a vape shop to a nearby business in a multiple-tenant retail building	Careen Khachatoorian, Peyton Jacob III, Neal L Benowitz, Prue Talbot [51]	2018	USA	N/A	Cotton towel, paper towel, terry cloth and two air filters	Nicotine	N/A	Lc–MS/MS Environmental assessments
Exposure and Risk Assessment of Second- and Third Hand Tobacco Smoke Using Urinary Cotinine Levels in South Korea	Jiyeon Yang, Shervin Hashemi, Wonseok Han, Yoojin Song and Youngwook Lim [34]	2022	Korea	Individuals living in South Korea who are non-smokers or former smokers living with at least one smoker in the same family	Urine, questionnaire	Cotinine	3203	Standardized laboratory techniques such as enzyme-linked immunosorbent assay (ELISA) or liquid chromatography–mass spectrometry (LC–MS)/ Human biological samples assessment, epidemiological assessment
Hand nicotine as an independent marker of thirdhand smoke pollution in children’s environments	E. Melinda Mahabee-Gittens, Ashley L. Merianos, Lara Stone, Chase A. Wullenweber, Penelope J.E. Quintana, Eunha Hoh, Nathan G. Dodder, Nicolas Lopez-Galvez, Georg E. Matt [13]	2022	USA	0–11 yrs old	Hand wipes/urine	Nicotine (parent compound), cotinine and 3HC	175	Liquid chromatography–tandem mass spectrometry (LC–MS/MS), questionnaires/ Human biological samples assessment, epidemiological assessment
Handwashing Results in Incomplete Nicotine Removal from Fingers of Individuals who Smoke: A Randomized Controlled Experiment	Thomas F. Northrup, Angela L. Stotts, Robert Suchting, Amir M. Khan, Michelle R. Klawans, Charles Green, Eunha Hoh, Melbourne F. Hovell, Georg E. Matt, Penelope J. E. Quintana [35]	2021	USA	Adults who smoke and visit infants in neonatal intensive care units (NICUs)	Hand wipes and skin	Nicotine	14	After participants washed or sanitized their hands, three separate fingers (thumb, index, and middle) were wiped at various time points to assess the efficacy of handwashing and sanitization in removing finger nicotine residue/ Human biological samples assessment
Identification and determination of the volatile organics of third-hand smoke from different cigarettes and clothing fabrics	Elahe Tondro Borujeni, Kamyar Yaghmaian, Kazem Naddafi, Mohammad Sadegh Hassanvand, Maziar Naderi [50]	2021	Iran	N/A	Clothing fabrics such as cotton, wool, polyester, and filament fabrics.	Benzene, toluene, xylene, pyridine, limonene, naphthalene, furfural, and nicotine	4	Solid phase microextraction (SPME) coupled with gas chromatography–mass spectrometry (GC–MS)/ Environmental assessments
Levels of 4-(methylnitrosamino)- 1-(3-pyridyl)-1-butanone (NNK) in raw wastewater as an innovative perspective for investigating population-wide exposure to third-hand smoke	Foon Yin Lai, Katerina Lympousi, Frederic Been, Lisa Benaglia, Robin Udrisard, Olivier Delémont, Pierre Esseiva, Nikolaos S. Thomaidis, Adrian Covaci and Alexander L. N. van Nuijs [60]	2018	Belgium	Individuals living in four European cities: Athens (Greece), Geneva (Switzerland), Geraardsbergen, and Ninove (both in Belgium)	Sewage	Cotinine and trans-3′-hydroxycotinine (3CH)	N/A	Liquid chromatography coupled with tandem mass spectrometry/ Environmental assessments
Medical staff contributions to thirdhand smoke contamination in a neonatal intensive care unit	Thomas F. Northrup, Angela L. Stotts, Robert Suchting, Amir M. Khan, Charles Green, Penelope J. E. Quintana, Eunha Hoh, Melbourne F. Hovell, Georg E. Matt [36]	2019	USA	Medical staff working in neonatal intensive care units (NICUs)	Fingers, survey	Nicotine	246	LC–MS/MS, collecting finger wipes, survey/ Human biological samples assessment
Metabolites of a Tobacco-Specific Lung Carcinogen in Children exposed to second-hand or thirdhand tobacco smoke in their homes	Janet L. Thomas, Hongfei Guo, Steven G. Carmella, Silvia Balbo, Shaomei Han, Andrew Davis, Andrea Yoder, Sharon E. Murphy, Larry C. An, Jasjit S. Ahluwalia, and Stephen S. Hecht [37]	2011	USA	Low-income families with children aged 10 or younger	Urine, questionnaire	Cotinine and nicotine	258	Liquid chromatography–electrospray ionization–tandem mass spectrometry (LC–ESI–MS/MS)/ Human biological samples assessment
Nicotine on children’s hands: limited protection of smoking bans and initial clinical findings	E Melinda Mahabee-Gittens, Ashley L Merianos, Eunha Hoh, Penelope JE Quintana and Georg E Matt [38]	2019	USA	Children who live in homes with smokers and are exposed to second-hand smoke	Hand wipes	Nicotine	104	Hand wipes and LC–MS/MS, questionnaire/ Human biological samples assessment, epidemiological assessment
Persistent tobacco smoke residue in multiunit housing: legacy of permissive indoor smoking policies and challenges in the implementation of smoking bans	Georg E. Matt, Penelope J.E. Quintanab, Eunha Hohb, Joy M. Zakarianc, Nathan G. Dodderc, Rachael A. Recordd, Melbourne F. Hovellb, E. Melinda Mahabee-Gittense, Samuel Padillac, Laura Markmanb, Kayo Watanabeb, Thomas E. Novotn [39]	2020	USA	Low-income multiunit housing residents	Surface wipes, survey	Nicotine	220	LC–MS/MS, gas chromatography–ion trap–tandem mass spectrometry (GC–IT–MS/MS)/ Human biological samples assessment, epidemiological assessment
Preliminary evidence that high levels of nicotine on children’s hands may contribute to overall tobacco smoke exposure	E Melinda Mahabee-Gittens, Ashley L Merianos, Georg E Matt [40]	2017	USA	Children	Saliva, hand wipes	Nicotine, cotinine	25	Liquid chromatography–tandem mass spectrometry (LC–MS/MS) or enzyme-linked immunosorbent assay (ELISA), gas chromatography–mass spectrometry (GC–MS) or high-performance liquid chromatography (HPLC)/ Human biological samples assessment
Remediating thirdhand smoke pollution in multiunit housing:temporary reductions and the challenges of persistent reservoirs	Georg E. Matt, Penelope J. E. Quintana, Eunha Hoh, Joy M. Zakarian, Nathan G. Dodder, Rachael A. Record, Melbourne F. Hovell, E. Melinda Mahabee-Gittens, Samuel Padilla, Laura Markman, Kayo Watanabe, Thomas E. Novotny [41]	2020	USA	Individuals living in low-income housing with elevated levels of thirdhand smoke	Dust samples collected from various surfaces in the participants’ homes	Nicotine	48	Liquid chromatography with triple quadrupole mass spectrometry (LC–MS/MS)/ Environmental assessments
Residual tobacco smoke in used cars: futile efforts and persistent pollutants	Addie L. Fortmann, Romina A. Romero, Marisa Sklar, Viet Pham, Joy Zakarian, Penelope J. E. Quintana, Dale Chatfield, Georg E. Matt [18]	2010	USA	Primary drivers of used cars—both smokers and non-smokers	Surface wipe, air, and dust samples collected from the interiors of cars, survey	Nicotine	127	Questionnaire/ Epidemiological assessment
Sources of tobacco smoke exposure and their associations with serum cotinine levels among us children and adolescents	Ashley L. Merianos, Timothy M. Stone, Roman A. Jandarov, E. Melinda Mahabee-Gittens, Kelvin Choi [42]	2022	USA	US school-aged children ages 6–11 years and adolescents ages 12–17 years	Serum, survey	Cotinine	5296	Isotope-dilution high-performance liquid chromatography–atmospheric pressure chemical ionization tandem mass spectrometric (ID HPLC–APCI–MS/MS), questionnaire/ Human biological samples assessment, epidemiological assessment
Thirdhand exposure at homes: Assessment using salivary cotinine	Cristina Lid’ on-Moyanoa, Marcela Fu, Raúl P’ erez-Ortuno, Montse Ballb, Esteve Garcia, Juan Carlos Martín-S’ anchez a, Jos A. Pascual, Esteve Fern’ andez, Jose M. Martínez-S’ anchez [43]	2020	Spain	Adults (≥16 years)	Saliva, questionnaire	Cotinine	519	Liquid chromatography coupled with tandem mass spectrometry (LC–MS/MS)/ Human biological samples assessment, epidemiological assessment
Thirdhand cigarette smoke in an experimental chamber: evidence of surface deposition of nicotine, nitrosamines and polycyclic aromatic hydrocarbons and de novo formation of NNK	Suzaynn F Schick, Kathryn F Farraro, Charles Perrino, Mohamad Sleiman, Glenn van de Vossenberg, Michael P Trinh, S Katharine Hammond, Bryan M Jenkins, John Balmes [52]	2013	USA	Individuals who are exposed to indoor environments where thirdhand smoke (THS)	Cotton cloth, paper	Nicotine, cotinine, tobacco-specific nitrosamines, and polycyclic aromatic hydrocarbons (PAHs)	N/A	Gc–MS/MS Environmental assessments
Thirdhand smoke and exposure in California hotels: non-smoking rooms fail to protect non-smoking hotel guests from tobacco smoke exposure	Georg E Matt, Penelope J E Quintana, Addie L Fortmann, Joy M Zakarian, Vanessa E Galaviz, Dale A Chatfield, Eunha Hoh, Melbourne F Hovell, Carl Winston [56]	2012	USA	Non-smokers who are exposed to tobacco smoke pollutants in hotel environment	Urine and finger wipe, survey	Cotinine	N/A	Liquid chromatography–tandem mass spectrometry (LC–MS/MS) with electrospray ionization (ESI). Isotope-dilution mass spectrometry (IDMS) techniques were utilized to quantify the concentrations of metabolites present in the samples/ Human biological samples assessment, epidemiological assessment
Thirdhand smoke causes DNA damage in human cells	Bo Hang, Altaf H. Sarker, Christopher Havel, Saikat Saha, Tapas K. Hazra, Suzaynn Schick, Peyton Jacob III, Virender K. Rehan, Ahmed Chenna, Divya Sharan, Mohamad Sleiman, Hugo Destaillats and Lara A. Gundel [54]	2013	USA	Individuals who are exposed to indoor environments where thirdhand smoke (THS)	Cellulose substrates	Nicotine and tobacco-specific nitrosamines (TSNAs)	N/A	LC–MS/MS, gas chromatography–ion trap–tandem mass spectrometry (GC–IT–MS/MS)/ Cellular analysis
Thirdhand smoke contamination and infant nicotine exposure in a neonatal intensive care unit: an observational study	Thomas F. Northrup, Angela L. Stotts, Robert Suchting, Amir M. Khan, Charles Green, Michelle R. Klawans, Penelope J. E. Quintana, Eunha Hoh, Melbourne F. Hovell, Georg E. Matt [45]	2020	USA	Metropolitan neonatal intensive care unit (nicu), including both visitors to the nicu and mother–infant dyads being cared for in the unit	Finger-nicotine wipes, furniture-nicotine wipes from mother–infant dyads, infant urine samples, interview	Cotinine	311	Liquid chromatography–tandem mass spectrometry (LC–MS/MS), interview/ Human biological samples assessment, epidemiological assessment
Thirdhand smoke contamination in hospital settings: assessing exposure risk for vulnerable paediatric patients	Thomas F Northrup, Amir M Khan, Peyton Jacob III, Neal L Benowitz, Eunha Hoh, Melbourne F Hovell, Georg E Matt, Angela L Stotts [45]	2016	USA	Mothers who smoke and have infants in the neonatal ICU (NICU)	Urine, surface nicotine samples from various sources such as the participants’ fingers, the infants’ crib/incubator, and hospital-provided furniture, questionnaire	Nicotine, cotinine, trans-3′-hydroxycotinine (3HC), and 4-(methylnitrosamino)-1-(3-pyridyl)-1-butanol (NNAL)	5	Liquid chromatography–tandem mass spectrometry (LC–MS/MS)/ Human biological samples assessment, epidemiological assessment
Thirdhand smoke exposure- Differences in smoke exposure indices and cultural norms between hotels and motels in South Korea	Myung-Bae Park, Tae Sic Lee, Jee Eun Oh, Do Hoon Lee [46]	2021	Korea	Hotel and motel guests	Urine	Cotinine	28	Liquid chromatography–tandem mass spectrometry/ Human biological samples assessment
Thirdhand tobacco smoke: a tobacco-specific lung carcinogen on surfaces in smokers’ homes	Janet L. Thomas, Stephen S. Hecht, Xianghua Luo, Xun Ming, Jasjit S. Ahluwalia, Steven G. Carmella [57]	2013	USA	Areas where people smoke indoors, such as homes or indoor public spaces, where thirdhand tobacco smoke residues might accumulate	Surfaces or dust in indoor environments, questionnaire	NNAL is a biomarker for NNK exposure and was detected in the urine of individuals exposed to NNK	56	Liquid chromatography–tandem mass spectrometry (LC–MS/MS)/ Human biological samples assessment, epidemiological assessment
Towards smoke-free cars in the Republic of Korea: Evidence from environmental and biochemical monitoring of third- hand smoke exposure in taxis	Eun Young Park, Min Kyung Lim, Sun Yeol Hong, Jee Eun Oh, Bo Yoon Jeong, E Hwa Yun, Wonho Yang, Do-Hoon Lee [58]	2018	Korea	Taxi drivers and their passengers	Air and dust, questionnaire	NNK and nicotine	17	Passive samplers were used to collect air samples, gas chromatography and liquid chromatography/ Environmental assessments
When smokers move out and non-smokers move in residential thirdhand smoke pollution and exposure	Georg E Matt, Penelope J E Quintana, Joy M Zakarian, Addie L Fortmann, Dale A Chatfield, Eunha Hoh, Anna M Uribe, Melbourne F Hovell [25]	2010	USA	Smokers and non-smokers	Urine, finger wipes, questionnaire	Cotinine, nicotine	N/A	Liquid chromatography–tandem mass spectrometry (LC–MS/MS), surface wipes, surveys/ Human biological samples assessment, epidemiological assessment
Wipe sampling for nicotine as a marker of thirdhand tobacco smoke contamination on surfaces in homes, cars, and hotels	Penelope J. E. Quintana, Georg E. Matt, Dale Chatfield, Joy M. Zakarian, Addie L. Fortmann, Eunha Hoh [59]	2013	USA	N/A	Surface wipe	Nicotine	150	Liquid chromatography–tandem mass spectrometry (LC–MS/MS) (isotope-dilution mass spectrometric (IDMS) techniques used nicotine-d4 to quantify the nicotine concentrations)/ Environmental assessments

N/A denotes Not applicable, NNA denotes 4-(N-methyl-N-nitrosamino)-4-(3-pyridyl)butanal (a tobacco-specific nitrosamine), NNK denotes nicotine-derived nitrosamine ketone (4-(methylnitrosamino)-1-(3-pyridyl)-1-butanone, NNN denotes N′-nitrosonornicotine.

### 3.2. Evaluation of Exposed Matrices or Samples

To analyse biomarkers, less invasive biological matrices such as saliva, skin, or urine are used. The choice of matrix or sample type can provide a more comprehensive evaluation of biomarker exposure. The majority of the utilized biological samples in this review are urine, hand wipes and finger wipes and saliva samples. Environmental samples are included in this review such as surfaces, clothes, different types of fabrics and air. To analyse the epidemiological aspects, studies provided the surveys, questionnaires, interviews or used machine learning to collect the necessary data for measuring the amount of tobacco residues on human. To evaluate thirdhand smoke (THS) in human cells and the damage caused by tobacco products, studies have reported using cellulose papers. The samples are exposed to THS, and biomarkers are measured on them to assess the level of exposure and damage.

### 3.3. Human Biological Samples

The biological samples extracted from the selected studies included urine, saliva, hand/finger wipes, and serum. These non-invasive matrices can be utilized for the assessment of nicotine and its metabolites in human biological samples.

Twelve studies analysed urine samples from both adults and children to measure exposure to thirdhand smoke (THS) [13,25,31,32,33,34,37,44,45,46,55,56]. Additionally, hand/finger wipes were evaluated in 10 studies [13,25,31,33,35,36,38,40,44,56], saliva samples in 2 studies [40,43], and serum samples in 1 study [42] for measuring THS exposure.

### 3.4. Environmental Assessment

Researchers have evaluated nicotine exposure from tobacco heating systems (THS) across different environments, including indoor spaces (such as homes and multiunit residences), outdoor areas, used vehicles, and various surfaces like fabrics and furniture. To detect tobacco product metabolites, they have employed methods such as dust wipes (surface wipes), air sampling, and fabric analysis. This review categorizes these samples into three groups: [1] surfaces, [2] fabrics, and [3] sewage.

A total of nine studies focused on collecting surface dust wipes or air samples to measure the levels of nicotine, cotinine, or 3-hydroxycotinine (3HC) [18,32,39,41,44,45,57,58,59,61,62]. Furthermore, seven studies investigated THS nicotine exposure on various fabric types, including terry cloth, carpets, wool, cotton, clothing fibres, and upholstery materials [18,47,48,49,50,51,52]. In addition, one study analysed and measured THS in sewage systems [60].

### 3.5. Epidemiological Assessment

To the epidemiological aspects, the studies employed a variety of methods: 9 used questionnaires [25,33,34,37,38,43,45,53,57,58], 6 utilized surveys [18,31,36,39,42,56], 1 employed machine learning techniques [55], and 1 conducted interviews [44]. These methods aimed to collect sociodemographic information about the participants or their parents to gain further insights.

### 3.6. Cellular Analysis

Nicotine product residues can cause cellular damage. To assess the effects of nicotine products on cells, two studies utilized cellulose paper and cellulose strips [53,54]. In these studies, samples were placed in indoor settings exposed to thirdhand smoke, and dust was collected on the cellulose materials.

## 4. Discussion

Nowadays, thirdhand smoke has become a public health concern. The nicotine absorption process is rapid, occurring through mucosal membranes or body fluids such as saliva. Once inhaled or absorbed through the skin, nicotine enters the bloodstream, is distributed throughout the body, and crosses the blood–brain barrier in seconds, exhibiting its psychoactive effects [63].

Several studies have indicated that nicotine and its metabolites can persist in the body for several hours. Additionally, residues from tobacco products can be detected in house dust, air, and on both indoor and outdoor furniture. The half-life of nicotine in various body organs ranges from 1 to 2 h [64]. In contrast, its metabolites, such as cotinine and 3-hydroxycotinine (3HC), exhibit greater stability and possess longer half-lives, typically ranging from 16 to 19 h [63].

This review aims to evaluate various biological and non-biological samples, as well as assessment methods for thirdhand smoke in humans. The samples have been categorized into four distinct categories to measure the presence of thirdhand smoke in humans. Biofluids play a crucial role in detecting biomarkers such as cotinine and 3-hydroxycotinine (3HC) in the human body. They can be collected easily through non-invasive and safe methods, making them suitable for measuring nicotine metabolites and assessing exposure to thirdhand smoke (THS) [26]. Many of the studies that employed biofluid assessments also utilized questionnaires, surveys, or interviews to gather information on the sociodemographic characteristics and smoking statuses of both participants and their household members. Sociodemographic factors play a vital role in children’s exposure to tobacco smoke (THS). For instance, a study by Mahabee-Gittens et al. demonstrated that children residing in smoking households exhibited significantly higher levels of nicotine exposure compared to those living with non-smokers [13]. In this study, the hand wipes sampling method was able to detect nicotine levels as low as 0.19 ng per wipe using liquid chromatography–tandem mass spectrometry (LC–MS/MS) [13].

However, this sampling technique has certain limitations, including lower precision compared to other biological samples. For example, urine testing provides a measure of systemic exposure to tobacco smoke, reflecting both inhalation and dermal absorption, making it useful for assessing overall exposure levels [28]. Smoking, whether in indoor or outdoor environments, can lead to exposure to nicotine metabolites and thirdhand smoke. Residual nicotine and its metabolites can persist on a variety of surfaces, including furniture, vehicles, and textiles such as cotton and clothing. These residues remain biologically active long after smoking has ceased, adhering to surfaces effectively [20]. Moreover, dust particles serve as a significant medium for the accumulation and retention of nicotine and other harmful chemicals, contributing to their extended persistence in the environment [65]. High concentrations of nicotine, cotinine, and 3-hydroxycotinine (3HC) can adhere to fabric fibres in materials such as cotton, wool, carpet, and clothing, persisting for months after smoking has ceased [20]. This adhesion is a significant factor in the persistence of thirdhand smoke on furniture and other indoor surfaces long after active smoking has stopped [66]. The absence of a standardized protocol for sample collection and preparation further complicates the process. Despite these challenges, the method remains a valid and reliable tool for assessing THS exposure and associated health risks [59].

To analyse the impact of thirdhand smoke (THS) on cellular structures, researchers have employed cellulose paper and other substrates to detect THS in air and dust samples. Cellulose-based materials, including cellulose paper, possess the capacity to adsorb and remove nicotine and various metabolites from cigarette smoke [67]. This suggests that cellulose-based materials may be effective for the detection and characterization of THS, which primarily comprises these residual compounds [67]. Cellulose materials, particularly cellulose paper, are frequently utilized in studies focusing on THS detection due to their efficient adsorption and retention of residual tobacco smoke components and measure the toxicity of those components on cells [67].

In all assessment methods, sampling plays a crucial role in measuring thirdhand smoke (THS). Accurate sampling ensures more reliable measurements and improves the detection of the presence, concentration, and impact of THS. Employing other assessment techniques such as chromatography, with or without mass spectrometry, enhances the accuracy of results when determining THS concentrations in various samples. Various assessment methods, including gas chromatography–mass spectrometry (GC–MS) and liquid chromatography–tandem mass spectrometry (LC–MS/MS), have been utilized to measure and detect thirdhand smoke in a variety of samples [68]. These analytical techniques provide high sensitivity and specificity for quantifying nicotine and its metabolites in complex matrices such as biological fluids and environmental samples [68]. Furthermore, these methods can identify and confirm target analytes, which is essential for the accurate quantification of nicotine, cotinine, and 3-hydroxycotinine (3HC) in the samples [69]. GC–MS and LC–MS/MS methods can attain extremely low limits of detection and quantification, reaching the ng/mL range [68,69]. This level of sensitivity is essential for evaluating low-level exposures to nicotine and its metabolites. Chromatography (both gas and liquid) combined with mass spectrometry

Nicotine, cotinine, and 3HC are well-established biomarkers of tobacco smoke exposure, and their quantification using GC–MS and LC–MS/MS is crucial for evaluating tobacco use and thirdhand smoke exposure across different populations and environments [68,69].

This systematic review enhances our understanding of various assessment methods employed for detecting tobacco product residues and their effects on human health.

Additionally, the review identifies certain limitations, including the exclusion of studies published in languages other than English, which may influence the overall findings.

It is also important to note that diseases, such as renal disorders, can affect the accuracy of results obtained from human biological samples. Furthermore, dust or air samples may demonstrate reduced accuracy in environments with high levels of pollutants, potentially resulting in elevated concentrations of nicotine and other metabolites.

## 5. Conclusions

This systematic review has elucidated various categories of samples that can be measured and evaluated using selective testing methods frequently employed in the assessment of nicotine thirdhand smoke exposure. The studies analysed encompassed diverse sample types to gauge nicotine residue exposure across different populations and environments. As a result of this review, the samples used to evaluate nicotine residues in humans were classified into four categories: environmental samples, cellular samples, human biological samples, and epidemiological samples. All samples were analysed using gas chromatography–mass spectrometry (GC–MS) and liquid chromatography–tandem mass spectrometry (LC–MS/MS), both of which offer high sensitivity and specificity for quantifying tobacco products in various matrices.

Among the studies reviewed, human biological and environmental samples were predominantly utilized to assess nicotine thirdhand smoke exposure. Studies employing human biological samples frequently conducted epidemiological assessments, such as questionnaires and surveys, to gather detailed information regarding the sociodemographic characteristics of participants. Notably, many studies included in this review were characterized by small sample sizes, which may impose limitations on the generalizability and reliability of the findings. Therefore, it is imperative to validate the conclusions of this review through future research involving larger sample sizes with standardized protocols, underscoring the need for further investigation to confirm these results.

## Data Availability

The original contributions presented in this study are included in the article/supplementary material. Further inquiries can be directed to the corresponding author.

## Supplementary Materials

The following supporting information can be downloaded at: https://www.mdpi.com/article/10.3390/ijerph22040621/s1, Table S1: ProQuest (Ovid) search table, Table S2: Scopus search table, Table S3: Medline (Ovid) search table, Table S4: Embase (Ovid) search table, Table S5: Cochrane library search table, and Table S6: Data extraction sheet template.

## Abbreviations

SHS	Second-hand smoke
THS	Thirdhand smoke
3HC	3-hydroxycotinine
PROSPERO	Prospective Register of Systematic Reviews
PRISMA	Preferred Reporting Items for Systematic Reviews and Meta-Analyses
LC–MS/MS	Liquid chromatography–tandem mass spectrometry
GC–MS	Gas chromatography–mass spectrometry
