# Supplementary material for: Analysis of Assessment Methods for Detecting Nicotine Residue and Its Impact on Humans: A Systematic Review
Source: Int J Environ Res Public Health. 2025 Apr 16;22(4):621. doi: 10.3390/ijerph22040621 (PMC12027364; doi:10.3390/ijerph22040621)
Supplement: Supplementary file 1 [file ijerph-22-00621-s001.zip › ijerph-3439941-supplementary.pdf]

Table S1. ProQuest (Ovid) search table

Group	Search syntax	Number of articles
1	Abstract(third-hand OR thirdhand OR ths) AND title((third-hand OR thirdhand OR ths))	403
2	Abstract(tobacco OR nicotin* OR ("volatile organic")) AND title((tobacco OR nicotin* OR ("volatile organic")))	168,854
3	Abstract(smok* OR product*) AND title((smok* OR product*))	1,486,249
4	Abstract(contamin* OR residue OR exposure OR pollut*) AND title((contamin* OR residue OR exposure OR pollut*))	931,299
5	Abstract(test* OR assess* OR detect* OR identif*) AND title((test* OR assess* OR detect* OR identif*))	3,722,977
6	1 & 2 & 3 & 4 & 5	7

(Date filtered: January 1999- September 2024)

Table S2. Scopus search table

Group	Search syntax	Number of articles
1	KEY ((third-hand OR thirdhand OR ths)) AND PUBYEAR > 1998 AND PUBYEAR < 2025	345
2	TITLE-ABS ((third-hand OR thirdhand OR ths)) AND PUBYEAR > 1998 AND PUBYEAR < 2025	4,135
3	1 & 2	266
4	KEY ((tobacco OR nicotin* OR "volatile organic*")) AND PUBYEAR > 1998 AND PUBYEAR < 2025	401,595
5	TITLE-ABS ((tobacco OR nicotin* OR "volatile organic*")) AND PUBYEAR > 1998 AND PUBYEAR < 2025	255,460
6	4 & 5	176,705
7	KEY ((smok* OR product*)) AND PUBYEAR > 1998 AND PUBYEAR < 2025	2,578,874
8	TITLE-ABS ((smok* OR product*)) AND PUBYEAR > 1998 AND PUBYEAR < 2025	6,139,870
9	7 & 8	1,794,000
10	KEY ((contamin* OR residue OR exposure OR pollut*)) AND PUBYEAR > 1998 AND PUBYEAR < 2025	1,610,565
11	TITLE-ABS ((contamin* OR residue OR exposure OR pollut*)) AND PUBYEAR > 1998 AND PUBYEAR < 2025	2,906,176
12	10 & 11	1,093,862
13	KEY ((test* OR assess* OR detect* OR identif*)) AND PUBYEAR > 1998 AND PUBYEAR < 2025	8,155,195
14	TITLE-ABS (test* OR assess* OR detect* OR identif*) AND PUBYEAR > 1998 AND PUBYEAR < 2025	21,706,426
15	13 & 14	6,065,939
16	3 & 6 & 9 & 12 & 15	16

(Date filtered: January 1999- September 2024)

Table S3. Medline (Ovid) search table

Group	Search syntax	Number of articles
1	(third-hand or thirdhand or ths).ti,ab.	2,650
2	(tobacco or nicotin* or "volatile organic").ti,ab.	227,082
3	(smok* or product*).ti,ab.	2,542,270
4	(contamin* or residue or exposure or pollut*).ti,ab.	1,628,558
5	(test* or assess* or detect* or identif*).ti,ab.	119,718,01
6	1 & 2 & 3 & 4 & 5	154
7	limit 6 to yr="1999 - 2024"	154

(Date filtered: January 1999- September 2024)

Table S4. Embase (Ovid) search table

Group	Search syntax	Number of articles
1	(third-hand or thirdhand or ths).ti,ab.	3,962
2	(tobacco or nicotin* or "volatile organic").ti,ab.	285,398
3	(smok* or product*).ti,ab.	3,246,836
4	(contamin* or residue or exposure or pollut*).ti,ab.	2,080,633
5	(test* or assess* or detect* or identif*).ti,ab.	16,205,465
6	1 & 2 & 3 & 4 & 5	197
7	limit 6 to yr="1999 - 2024"	197

(Date filtered: January 1999- September 2024)

Table S5. Cochrane library search table

Group	Search syntax	Number of articles
1	(third-hand or thirdhand or ths):ti,ab,kw	157
2	(tobacco or nicotin* or "volatile organic"):ti,ab,kw	17,739
3	(smok* or product*):ti,ab,kw	139,519
4	(contamin* or residue or exposure or pollut*):ti,ab,kw	95,132
5	(test* or assess* or detect* or identif*):ti,ab,kw	1,031,676
6	#1 and #2 and #3 and #4 and #5	41

(Date filtered: January 1999- September 2024)

Table S6. Data extraction sheet template

Title of the study	Author(s)	Year of publication	Country	Target population	Biological or non-biological samples	Biomarkers	Number of participants	Category of assessment method (s)
